# Unicentric Castleman’s disease in the parotid gland associated with psoriasis: a case report

**DOI:** 10.1186/s13256-024-04468-5

**Published:** 2024-04-03

**Authors:** Ying Zhang, Chong-Yang Li, Zhi Li, Wei Chen

**Affiliations:** Department of Stomatology, Jinling Hospital, Affiliated Hospital of Medical School, Nanjing University, 305 East Zhongshan Road, Nanjing, 210012 Jiangsu China

**Keywords:** Castleman’s disease, Interleukin-6, Parotid gland, Psoriasis

## Abstract

**Background:**

Castleman’s disease is a rare lymphoproliferative disorder that is often misdiagnosed because of its untypical clinical or imaging features except for a painless mass. Besides, it is also difficult to cure Castleman’s disease due to its unclear pathogenesis.

**Case presentation:**

We present a Castleman’s disease case with diagnostic significance regarding a 54-year-old Chinese male who has a painless mass in his left parotid gland for 18 months with a 30-years history of autoimmune disease psoriasis. Computed tomography scan showed a high-density nodule with clear boundaries in the left parotid and multiple enlarged lymph nodes in the left submandibular and neck region. General checkup, the extremely elevated serum interleukin-6 and lymph node biopsy in the left submandibular region gave us an initial suspicion of Castleman’s disease. Then the patient underwent a left superficial parotidectomy. Based on histopathologic analysis, we made a certain diagnosis of Castleman’s disease and gave corresponding treatments. In 18 months of follow-up, the patient showed no evidence of recurrence, with the level of serum interleukin-6 decreased.

**Conclusions:**

Clinicians should be aware of the possibility of Castleman’s disease when faced with masses or enlarged lymph nodes in the parotid gland to avoid misdiagnosis, especially in patients with autoimmune diseases and elevated serum interleukin-6.

**Supplementary Information:**

The online version contains supplementary material available at 10.1186/s13256-024-04468-5.

## Background

Castleman’s disease (CD) is a rare lymphoproliferative disorder, which has been described as giant lymph node hyperplasia, benign giant lymphoma, lymphoid hamartoma, and angiofollicular hamartoma due to its heterogeneous presentations [1]. Castleman’s disease can present in unicentric (UCD) and multicentric (MCD) forms [2]. UCD generally presents with an enlarging mass, without other significant symptoms, whereas MCD patients were more likely to share similar clinical characteristics with AID, like glomerulopathy, mucous lesions, and pulmonary complications [3]. For therapy, complete excision of the mass can be considered the gold standard of management for patients with UCD and can result in a 100% 5-year survival rate [4, 5].

Castleman’s disease can occur anywhere throughout the lymphatic system. The mediastinum is the most common location, followed by the head and neck, the abdomen, and the axilla [6]. However, to date, few cases of Castleman’s disease have been reported in the salivary gland, with an involvement rate of 6–14% [7]. Due to the lack of specific clinical features of this disease, it is difficult for stomatologists to recognize and diagnose Castleman’s disease of parotid in the early stage. Here, we report an unusual case of UCD located in the left parotid gland and present its clinical, imaging, and pathological findings.

## Case report

In November 2021, a 54-year-old male, Chinese patient consulted our hospital due to a painless swelling below his left temporal-mandibular joint for about 18 months, complaining that this mass gradually enlarged in the past 12 months. The patient worked freelance and was born and raised in Taizhou City, Jiangsu Province, China. He had a history of hypertension for 15 years. His blood pressure was under control with oral intake of irbesartan. Noteworthily, he also had a history of psoriasis for over 30 years without any treatments. After admission, the patient developed extensive red plaques covered with thick white scales. According to the suggestion of dermatologists, halomethasone and calcipotriol were given to relieve relevant skin symptoms. The patient has no history of smoking or drinking alcohol. Also, he has no history of infectious or genetic diseases.

At admission, the blood pressure of the patients was 145/90 mmHg, with a temperature of 36.5℃, a heart rate of 85 beats per minute, and a respiratory rate of 18 times per minute. He was generally well-developed and a good nutrition. He was alert with good cognitive function and was able to take a physical examination. His skin was warm and dry, with scattered rashes but no bleeding. His conjunctivae and sclerae were normal without jaundice. His pupils were equal round with normal reflexes. He had good eyesight without strabismus, nystagmus, or exophthalmos. His bilateral hearing was intact without ear deformity, discharge, or tenderness. His neck was supple without tenderness. His trachea was midline and his thyroid gland was normal. Jugular venous distension was not found. Pulmonary, cardiovascular, and abdominal examinations showed no abnormalities. Neurological examinations showed that the superficial, deep, and compound sensations are normal. The superficial, biceps brachii, and Achilles tendon reflex are normal. The physiological reflex is present, but the pathological reflex is not elicited. Kernig's syndrome is normal.

Physical examination showed a painless mass of about 2 × 3 cm in size with moderate hardness and a clear boundary in his left parotid gland. Multiple enlarged lymph nodes were palpated in the left submandibular and neck region, with different sizes from about 0.5 to 3 cm. No enlarged lymph node was palpated in the right corresponding region. The oral hygiene condition is acceptable, with no caries or missing teeth, no redness or swelling in the gums and mucosa, and no obvious abnormalities in the remains. The general check of his oral cavity excluded the cause of intraoral infection for enlarged lymph nodes.

**Fig. 1:**
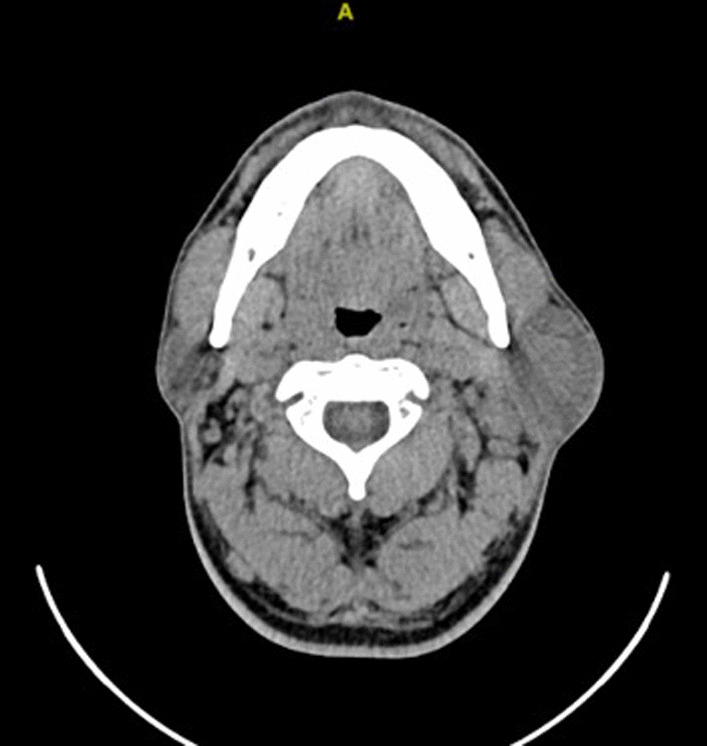
The axial cut in the Computer Tomography scan. The axial cut in the Computer Tomography scan of the head and neck, slightly high-density nodular shadows were shown at the left parotid gland

Computer Tomography (CT) scan showed a slightly high-density nodular mass in the left parotid gland, about 29 mm × 20 mm in size, and uniform in density. There were multiple enlarged lymph nodes on the medial border of the sternocleidomastoid muscle in the left submandibular region (Fig. 1). PET-CT of the whole body showed enlarged lymph nodes in the left parotid gland region, left neck, and left submandibular multiple with increased FDG metabolism. All results of laboratory findings are shown in Additional file 1: Table S1. Laboratory data showed Human immunodeficiency virus test (HIV) was negative. However, the level of serum interleukin (IL)-6 was extremely high (13.42 ng/L). To further clarify the diagnosis, after ruling out contraindications to surgery and anesthesia, the lymph node biopsy in the left submandibular region was performed under local anesthesia. The pathological report showed lymphoid tissue hyperplasia with vascular hyperplasia, considered as Castleman’s disease (hyaline-vascular type). Combined with his history of psoriasis, it suggested that his immune system was disordered and gave us an initial suspicion of Castleman’s disease.

Therefore, we planned to remove the tumor by superficial parotidectomy and clarify the diagnosis by pathological findings. The patient gave consent for this operation plan and underwent a left superficial parotidectomy with facial nerve perseveration under general anesthesia. The S-shaped incision was made, and the mass was excised with careful separation of the facial nerve to avoid damage. The removed mass has a gray-brown nodular appearance, and its cut surface was gray-white and soft. It was partially paginated, surrounded by a small amount of salivary gland tissue with clear boundaries. The whole tumor and the left superficial parotid gland were removed successfully without injury to facial nerves. The patient recovered well after the surgery, with no obvious complaints of discomfort.

**Fig. 2:**
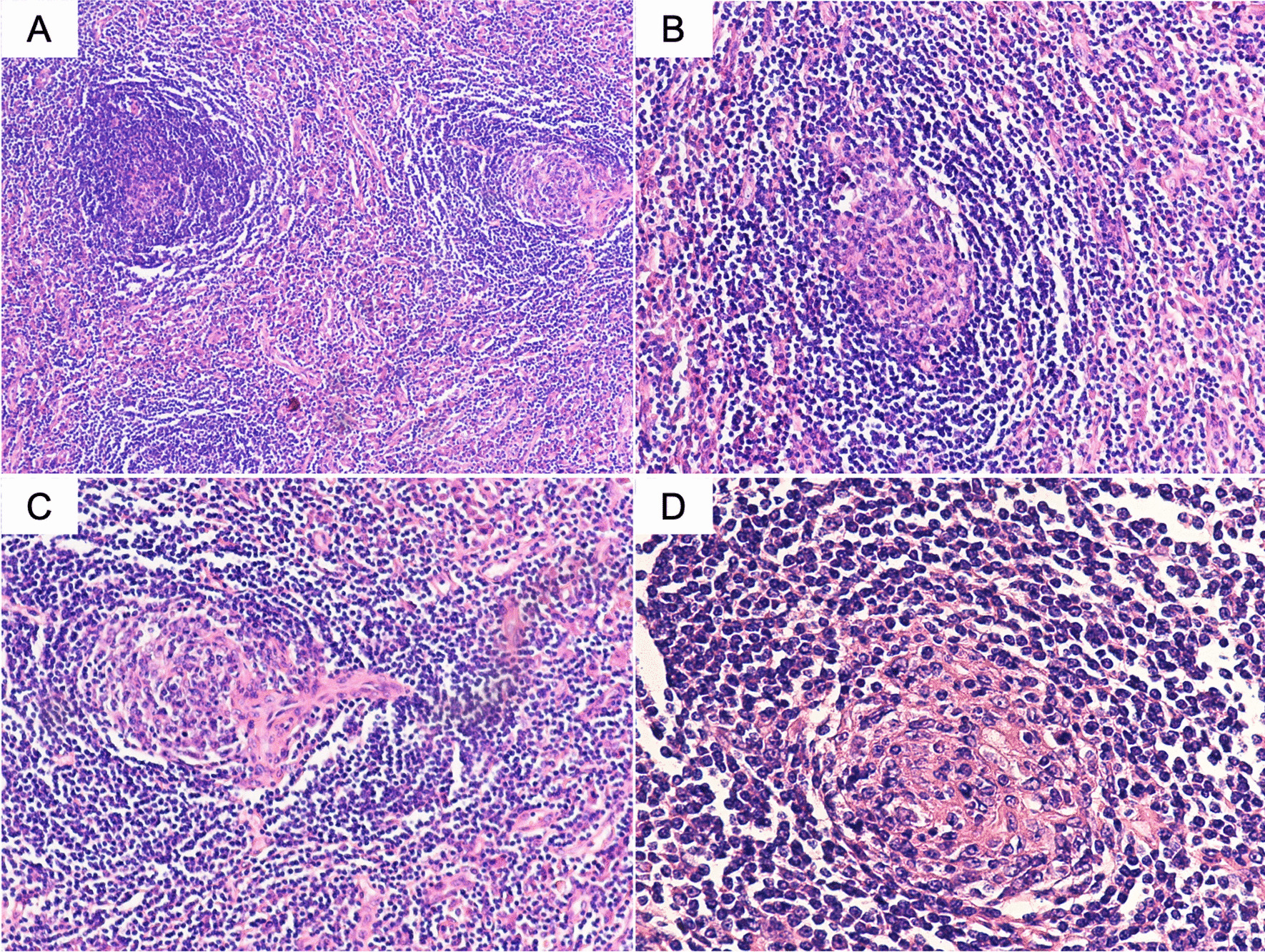
The histological microphotographs of Castleman’s disease (hematoxylin–eosin stain). **A** Low magnification of the lesion showed areas of vascular proliferation distributed in the interfollicular regions (original magnification × 100). **B** The lymphoid parenchyma was composed of the lymphoid follicle with the characteristic of “onion skin” laying of lymphocytes in the mantle zone (original magnification × 200). **C** The “lollipop” appearance was formed by radially penetrating sclerotic blood vessels with concentric mantle zones (original magnification × 200). **D** High magnification of mantle cell layers in targetoid with the vascular proliferation in germinal center (original magnification × 400)

The histological examination of the excised tumor showed the lymphoid follicle hyperplasia, the proliferation of capillaries with extensive hyaline degeneration, and some lymphocytes arranged in concentric circles surrounding the lymphoid follicles, thus like the appearance of “onion skin” layering in the mantle zone (Fig. 2A, B). In interfollicular sectors, multiple hyaline vascular structures were noticed, with the “lollipop” appearance, which was formed by radially penetrating sclerotic blood vessels with concentric mantle zones (Fig. 2C, D). The immunohistochemical study revealed that tumor cells were positive for CD20 + mainly in the follicular regions. B-cell lymphoma 2 (Bcl-2) was positive and T cells were positive for CD3. CD21 and CD35 showed the follicular dendritic cell network.

Based on H&E and immunohistochemical analysis, we made a certain diagnosis of Castleman’s disease (hyaline-vascular type) and gave corresponding postoperative care and treatments to this patient. During the hospital stay, the patient was intravenously administrated with Cefoxitin 1.0 g and Ornidazole 0.5 g per day for one week, 0.01 g Dexamethasone per day for three days. He was also treated with methylcobalamin tablet 1.5 mg per day until two weeks after discharge. In the 18 months follow-up, the patient maintained good health without recurrence or metastasis. The laboratory data showed the level of serum interleukin-6 was extremely decreased compared with that before surgery (8.05 ng/L).

## Discussion

We present an important CD case with diagnostic significance regarding a 54-year-old male who had a gradually growing and painless mass in his left parotid gland for 18 months. Notably, he had a history of psoriasis for over 30 years without any treatments. Although UCDs were reported to occur in patients with any other AIDs such as paraneoplastic pemphigus, myasthenia gravis, and so on, it is rarely encountered in the parotid gland associated with psoriasis [8]. Furthermore, preoperative examination showed that the serum IL-6 level was elevated. Then the patient underwent a left superficial parotidectomy, and H&E and immunohistochemical analysis gave us a certain diagnosis of Castleman’s disease (hyaline-vascular type). In the 18 months follow-up, he recovered well without recurrence and the level of serum IL-6 was significantly decreased compared with that before surgery.

Patients with UCD are often asymptomatic which may result in diagnosis delay in clinic. Actually, the epidemiology of UCD is poorly understood and risk factors for UCD remain unknown, owing to its low incidence (the total morbidity of Castleman’s disease is estimated at 21–25 per million person-years). In recent years, Castleman’s disease was often found to be complicated with autoimmune diseases (AID). However, the association between these two diseases was not fully illustrated due to limited studies [9]. It was supposed that the emergence of CD may be related to prolonged use of immunomodulatory therapy for the treatment of psoriasis [3]. Before, Mohagheghi *et al.* reported a 32-year-old psoriasis patient diagnosed with UCD, of whom received 4-years immunosuppressive treatment for psoriasis [10]. Sears *et al.* also presented a 50-yar-old man with severe chronic psoriasis and psoriatic arthritis, in whom Castleman’s disease arose after anti-TNF biologic therapy [11]. This is the first patient with UCD in the parotid gland reported to have a history of psoriasis. However, this still need more evidence from high quality epidemiological studies.

Another characteristic of this patient is the significantly elevated serum interleukin-6 level. It has been well-documented that interleukin-6 plays a critical role in the development of MCD [12]. Many symptoms related to MCD could also be attributed to interleukin-6 over-expression. Siltuximab, an anti- interleukin-6 monoclonal antibody, has been licensed by the FDA for the treatment of MCD, due to its proven effects in the initial trials [13]. However, interleukin-6 levels are not routinely tested in adult UCD patients because the presence of increased interleukin-6 levels is rare. The interleukin-6 expression of the patient we reported was significantly down-regulated after the superficial parotidectomy, which is consistent with that of the case reported by Lyapichev *et al.* [14]. This indicates that interleukin-6 could be the biomarker for the response to surgical treatments in these two patients. However, this result should be interpreted carefully, considering that the interleukin-6 levels were still higher than normal after the surgery. Normally, it takes some time for IL-6 to recover to normal levels, so a long-term follow-up with the regular monitor of the IL-6 level will help to further determine its prognostic significance for UCD.

## Conclusion

In summary, we reported a case of UCD with a history of psoriasis rarely encountered in the parotid gland. The longstanding psoriasis may be associated with the onset of UCD and the significantly down-regulated interleukin-6 level indicated a good therapeutic response. These findings suggest that detecting immune diseases and measuring interleukin-6 level could be helpful in the diagnosis and prognosis of UCD.

### Supplementary Information

**Additional file 1.** Results of laboratory findings.

## Data Availability

All data generated or analyzed during this study are included in this published article and its supplementary information files.

## Abbreviations

AID: Autoimmune diseases
Bcl-2: B-cell lymphoma 2
CD: Castleman’s disease
CT: Computer tomography
HIV: Human immunodeficiency virus test
IL: Interleukin
MCD: Multicentric Castleman’s disease
UCD: Unicentric Castleman’s disease
