# Supplementary material for: Unicentric Castleman’s disease in the parotid gland associated with psoriasis: a case report
Source: J Med Case Rep. 2024 Apr 3;18:140. doi: 10.1186/s13256-024-04468-5 (PMC10988862; doi:10.1186/s13256-024-04468-5)
Supplement: Supplementary file 1 — Additional file 1. Results of laboratory findings. [file 13256_2024_4468_MOESM1_ESM.docx]

**Table S1. Results of laboratory findings**

| Laboratory tests | Result | Reference range |
| --- | --- | --- |
| White blood cell count | 5.70×10^9/L | 3.5-9.5×10^9/L |
| Lymphocyte count | 1.99×10^9/L | 1.1-3.2×10^9/L |
| Monocyte count | 0.42×10^9/L | 0.1-0.6×10^9/L |
| Neutrophil count | 3.17×10^9/L | 1.8-6.3×10^9/L |
| Red blood cell count | 4.70×10^12/L | 4.3-5.8×10^12/L |
| Hemoglobin | 154 | 130-175 g/L |
| Platelet count | 348×10^9/L | 125-350×10^9/L |
| Calcium level | 2.28 | 2.11-2.52 mmol/L |
| Potassium level | 3.5 | 3.5-5.3 mmol/L |
| Sodium level | 144 | 137-147 mmol/L |
| Albumin level | 41.3 | 40-55 g/L |
| Globulin level | 24.4 | 20-40 g/L |
| CRP level | < 0.5 | 0-8 mg/L |
| ALP level | 50 | 45-125 U/L |
| GTP level | 27 | 10-60 U/L |
| Urea level | 6.1 | 3.1-8 mmol/L |
| Creatinine level | 74.7 | 57-97 𝜇mol/L |
| Uric acid level | 424 | 210-430 𝜇mol/L |
| Cystatin C level | 1.17 | 0.6-1.18 mg/L |
| IL-6 level | 13.42 | < 7 ng/L |
| Urinalysis | | |
| Protein count | 1 |  |
| Pus cell count | 1-2/HPF |  |
| Red cell count | 1/HPF |  |
| Urine culture | No growth |  |
| HIV antigen/antibody | Negative |  |

CRP C-reactive protein, ALP alkaline phosphatase, GTP glutamyl transpeptidase, IL-6 interleukin-6, HIV human immunodeficiency virus
